# Malaria chemoprophylaxis recommendations for immigrants to Europe, visiting relatives and friends - a Delphi method study

**DOI:** 10.1186/1475-2875-10-137

**Published:** 2011-05-20

**Authors:** Guido Calleri, Ron H Behrens, Matthias L Schmid, Federico Gobbi, Martin P Grobusch, Francesco Castelli, Joaquim Gascon, Zeno Bisoffi, Tomas Jelinek, Pietro Caramello

**Affiliations:** 1 Amedeo di Savoia Hospital, Department of Infectious Diseases, Travel Medicine Unit, Corso Svizzera 164, 10149-Torino, Italy; 2 Dept of Infectious and Tropical Diseases, London School of Hygiene and Tropical Medicine, Keppel St, London WC1E 7HT, UK; 3 Department of Infection & Tropical Medicine, Royal Victoria Infirmary, Queen Victoria Road, Newcastle upon Tyne, NE1 4LP, UK; 4 Centre for Tropical Diseases, Ospedale Sacro Cuore, via Don Sempreboni 5, 37024 Negrar (Verona), Italy; 5 Dept Infectious Diseases, Tropical Medicine and HIV/AIDS, Division of Medicine, Academic Medical Centrum (AMC), University of Amsterdam, Amsterdam, Netherlands; 6 Institute for Infectious and Tropical Diseases, University of Brescia, piazzale Spedali Civili 1, 25123 Brescia, Italy; 7 Barcelona Centre for International Health Research (CRESIB), Hospital Clinic/IDIBAPS, University of Barcelona, Spain. c/Villarroel, 170, 08036 Barcelona, Spain; 8 Centre for Tropical Diseases, Ospedale Sacro Cuore, via Don Sempreboni 5, 37024 Negrar (Verona), Italy; 9 Berlin Centre for Travel and Tropical Medicine, Jägerstr. 67-69, 10117 Berlin, Germany; 10 Infectious Diseases Unit, Amedeo di Savoia Hospital, ASL TO2, c. Svizzera 164, 10149 Torino, Italy

## Abstract

**Background:**

Numbers of travellers visiting friends and relatives (VFRs) from Europe to malaria endemic countries are increasing and include long-term and second generation immigrants, who represent the major burden of malaria cases imported back into Europe. Most recommendations for malaria chemoprophylaxis lack a solid evidence base, and often fail to address the cultural, social and economic needs of VFRs.

**Methods:**

European travel medicine experts, who are members of TropNetEurop, completed a sequential series of questionnaires according to the Delphi method. This technique aims at evaluating and developing a consensus through repeated iterations of questionnaires. The questionnaires in this study included questions about professional experience with VFRs, controversial issues in malaria prophylaxis, and 16 scenarios exploring indications for prescribing and choice of chemoprophylaxis.

**Results:**

The experience of participants was rather diverse as was their selection of chemoprophylaxis regimen. A significant consensus was observed in only seven of 16 scenarios. The analysis revealed a wide variation in prescribing choices with preferences grouped by region of practice and increased prescribing seen in Northern Europe compared to Central Europe.

**Conclusions:**

Improving the evidence base on efficacy, adherence to chemoprophylaxis and risk of malaria and encouraging discussion among experts, using techniques such as the Delphi method, may reduce the variability in prescription in European travel clinics.

## Background

For most of the twentieth century, immigration from malaria-endemic countries to Europe has been associated with relationship between European countries and their former colonies. Influx of immigrants has been variable over time influenced by geography, period of time, political stability and economic changes. Arrival of migrants to France, Spain, Belgium and the UK, has occurred over many decades because of their colonial links, but migration to Italy, Greece and Eastern European Countries has developed more recently with a much more ethnically heterogeneous group.

For example, because of language and cultural connections, Spain receives migrants from Latin America, France from northern and western Africa, the UK from Asian and Central East African Countries. Italy has recently become a destination for migrants from many regions in Africa, because of its proximity to North Africa.

Many immigrants integrate into their host society and have developed family roots, but frequently travel to their country of origin to visit friends and relatives (VFRs). There is now good evidence that this group of travellers is at increased risk of acquiring infectious diseases such as malaria, for a number of biological, behavioural and geographical reasons [1], whereas many of their children may be born in Europe and lack malaria immunity.

The proportion of VFRs who seek pre-travel advice is lower than in other groups of travellers. This and other factors such as their health beliefs, mode and place of travel and affordability of drugs and vaccines contribute to their increased risk of morbidity during travel [1]. On return, they present more frequently with severe, preventable infectious diseases than tourists [2-4]. In particular their risk of acquiring malaria appears significantly greater, compared to other travellers [5].

The practice of advising VFRs in pre-travel clinics varies throughout Europe, because of different health systems, immigrant populations and social support structure. In a previous study, using a Delphi approach in this group of experts, the policies of chemoprophylaxis recommendations in non-immune travellers varied widely among prescribers, influenced by a high degree of subjectivity and national policy differences [6].

The Delphi approach is a consensus development technique, which was introduced in 1952, to be used in situations where, in absence of scientific evidence, there is no unanimity of opinion [7-9]. Experts' views are explored to examine the range of policies followed in practice, through an iterative completion of questionnaires along with cumulative feedback [10]. The aim of this study was to investigate the opinions of major European experts, and to identify their degree of consensus when dealing with malaria prevention in VFRs.

## Methods

This study was undertaken in the framework of TropNetEurop, a European network of travel and tropical medicine centres, created to report cases of imported infections, exchange opinions and improve practice among professionals [11-13]. An initial study, focused on malaria chemoprophylaxis in all groups of travellers was published elsewhere [6]. In this paper, a follow-on study, designed to examine the experts' practices in advising VFRs travellers, is reported. Six experts in travel medicine, belonging to TropNetEurop and recognized as leaders in this field, prepared, discussed and designed the questionnaire. Following pilot testing the questionnaire was forwarded for completion to lead clinician in all network member sites (designated in the manuscript as "experts").

The first questionnaire (see additional file 1) examined the respondent's experience with VFRs, and problems encountered in prescribing malaria prophylaxis to this subset of travellers. Each question included a number of choices, and responses were based on a on a visual scale between 1 and 10. The responses were analysed as median and 1^st^-3^rd ^quartile difference. Some questions with a single numerical response were evaluated as means or medians.

The second part of the questionnaire investigated prescribing preferences for 16 travel scenarios (see additional file 2): participants chose their preferred chemoprophylaxis recommendations and gave reasons for this choice. Agreement was evaluated by the use of a homogeneity index for categorical variables, scoring 0 for no consensus (i.e. equal distribution throughout the three response choices: yes, no, uncertain) to 1 for complete consensus [14]. All results were captured using Microsoft Excel^©^. The survey was undertaken between May and September 2006.

## Results

Forty-seven questionnaires were distributed and 25 (53.1%) were returned and were evaluable. The geographic distribution of responding centres reflected the distribution of TropNetEurop centres (five in Germany, four in Italy, two in Denmark, Portugal, Spain and Switzerland, one each in Belgium, Finland, France, Ireland, Norway, Poland, Sweden and the UK).

**Figure 1:**
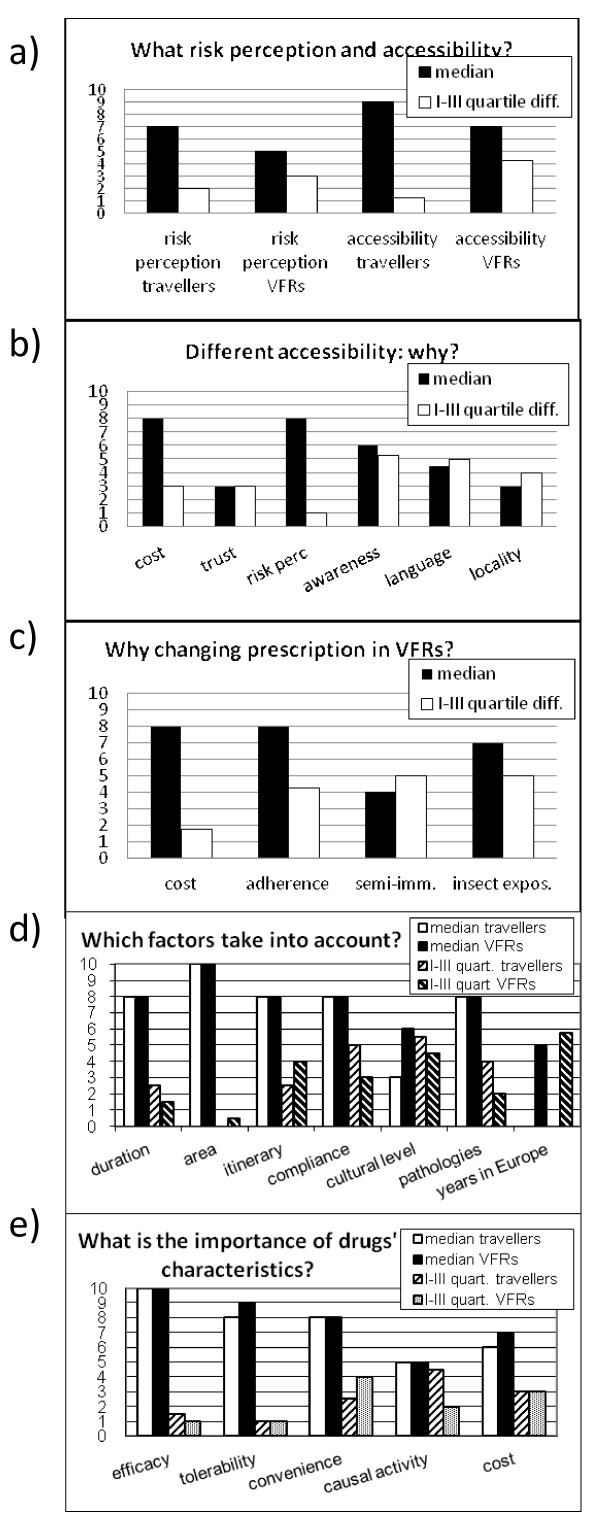
**Questionnaire section A: scores given by the experts to questions**. Median and interquartile differences of scores given by the experts to questions n. 1, 2, 3, 8, 10, 11 (see additional file 1). In graphs d) and e), the answers were compared to what the same experts responded to a previous questionnaire on non-VFR travellers [6].

VFR travellers make up a minority of attendees at most sites surveyed (less than 15% in 19 clinics and more than 35% in only two, Paris and Copenhagen). The majority of VFRs originate from West Africa, and the remainder from other parts of Africa. Only a minority were from the Indian Sub Continent or Latin America. Responses of experts to most questions are shown as median and interquartile differences in Figure 1.

The majority of respondents (17/25) recommended different prophylaxis regimens for VFRs than for other travellers, but 12 respondents would use the same recommendation after the immigrant has been resident for one to five years in the host country. Half of the experts (13/25) modified their advice where the traveller was accompanied by children. The proportion of VFR patients admitted with malaria on return from travelling was 43% of all malaria admissions (median; range: 10-85%).

**Figure 2:**
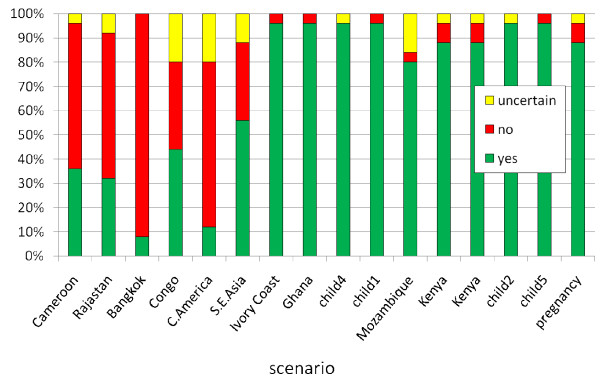
**Questionnaire section B: Responses of expert to single scenarios**. For everyone of the 16 scenarios the proportion of experts recommending prophylaxis (green bar) not recommending prophylaxis (red bar) or uncertain (yellow bar) is reported. For details about the scenarios, see additional file 2.

The results of the second part of the questionnaire (scenarios) are shown in Figure 2. Experts' affirmative responses to chemoprophylaxis across the 16 scenarios ranged between three and 15. Prescribers from Northern Europe gave an affirmative response in 12.5 scenarios (mean), while those from Central Europe only in 9.4. The difference at Student's t test is significant (p = 0.02). Prescribers from Southern Europe answered yes in 11.5 cases (non-significant versus other groups).

**Figure 3:**
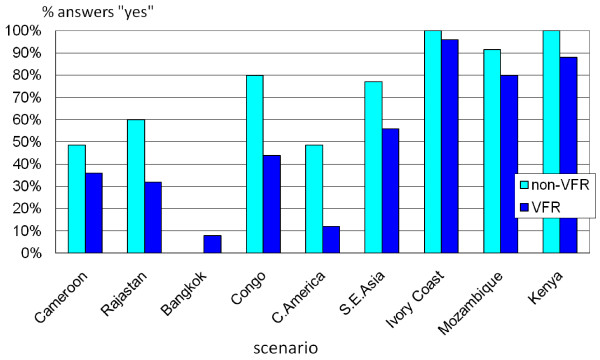
**Prophylaxis prescription in the proposed scenarios: non-VFRs and VFRs**. Comparison between scenarios referred to non-VFRs (in a previous questionnaire [6]) and VFRs. Only 9 similar scenarios and only answers by experts who have responded to both questionnaires are included.

Nine of the scenarios were very similar to those examined by the experts in the previous study and now referred to as non-VFRs [6]. A comparison of the recommendations given to VFRs and non-VFRs on the same scenarios is shown in Figure 3.

## Discussion

This study was a follow on from a previous study [6] investigating European experts' opinions on malaria prophylaxis in all travellers. In that study as well as in the present study, the experts were TropNetEurop centre managers: 19 experts took part in both studies. Although VFR travellers to different parts of the world have different malaria risk profiles, the first part of the questionnaire explored the philosophy when advising and examined differences in advice to VFRs and non-VFR travellers, but did not account for the heterogeneity of these populations due to other risk factors including destination and age. This aspect was explored in the second part of the questionnaire, where specific situations were examined. We identified variability in opinions, relating to numbers of VFRs seen in clinic, country or origin of this population and usage of travel clinic services. This probably reflects the varied national health systems regulations and country-specific history of immigration. Although VFRs make up a significant burden of imported malaria throughout Europe [15], they appear to represent a low proportion of travel clinic clients. This can be interpreted in a number of ways. They may reflect that this group is a low proportion of all travellers, but has increased malaria risk. VFRs may not perceive themselves to at risk of malaria and therefore not seek advice: a few studies have described VFRs malaria risk perception and use of prophylaxis when returning to their home countries [16-18]. VFRs may encounter barriers such as lack of information of services, language, trust of health systems, concerns on their legal status, and cost, which may limit their access to travel clinics [2]. Cost benefit modelling of the financial benefit of subsidising chemoprophylaxis for VFRs in France [19] and Switzerland [20] suggests that such a subsidy by the health/insurance provider is cost effective and would save money. Overcoming barriers encountered by VFRs would require combined efforts of professionals, policy makers, leaders in the communities and support from non-profit charity organizations. Factors that influence prophylaxis prescribing by experts were similar for tourists and VFRs (geographic area, duration, itinerary, compliance), but educational level was considered important when prescribing for the latter.

There was no consensus among experts regarding modifying chemoprophylaxis regimens for their VFR client, but the majority attempted to reduce cost and improve adherence. There are significant costs variations among regimens and for VFRs, who often have low income and stay abroad for longer, choosing the least expensive was an important option and influenced prescribing. There was no consensus on when an immigrant becomes more vulnerable or can be considered as a non-immune. Some authors suggest that 'semi-immunity' may last longer (more than four or five years) than has been commonly assumed [21-23]. Mascarello and colleagues report that settled migrants not exposed to malaria for more than eight years develop more severe malaria than those recently arrived [24]. Some respondents altered their prescribing to VFRs when they are accompanied by children (who are likely to be non-immune), in order to provide a single regimen for the whole family, and attempt to improve adherence.

The scenarios highlighted diverse chemoprophylaxis prescribing among experts by geographic regions within Europe. Northern European experts were more likely to recommend prophylaxis than Central Europeans. This may reflect differences of national guidelines, training, availability of drugs and experience of VFR travellers.

Comparing nine similar scenarios used in the initial survey [6] the differences in prophylaxis recommendations between VFRs and non-VFRs can be observed. Chemoprophylaxis was recommended less frequently for VFRs (in eight out of nine scenarios), and uncertainty in prescribing was more frequently expressed in this group (9.3% in VFRs vs. 5.8%). Explanations for this would include affordability of drugs and expected lack of adherence to prophylaxis.

**Figure 4:**
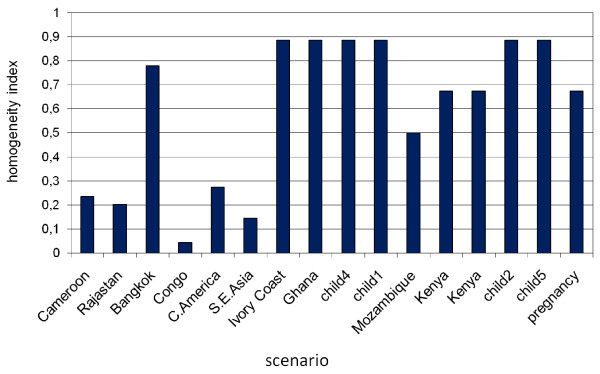
**Homogeneity index in answers proposed by experts to the 16 scenarios**. The homogeneity index for categorical variables ranges from 0 (no consensus, i.e equal distribution throughout the three response choices: yes, no, uncertain) to 1 (complete consensus on one choice). For details about the scenarios, see additional file 2.

Overall the study found a very low consensus in 5/16 scenarios and a low consensus, in a further 4/16 scenarios. Insufficient consensus in more than half of the scenarios suggests that opinion rather than evidence was used when deciding risk and indications, and highlights a deficit of evidence. Good evidence would also improve consistency of guidelines across Europe (Figure 4).

## Conclusions

VFRs have the highest proportion of malaria morbidity: addressing their needs and requirements is a priority. This study supports findings from the previous study within the same group of experts. There is a considerable variation in recommendations for malaria prophylaxis among professionals in Europe, both from a conceptual perspective, and in prescribing practice when assessed though responses to clinical scenarios. This may be due to insufficient detail provided in the scenarios or may be related to the heterogeneity of national guidelines across Europe. The most likely explanation is poverty of evidence on efficacy, adherence to medication and malaria risk faced by travellers, and in particular, evidence pertaining to VFRs. This lack of evidence results in recommendations largely based on personal opinion, particularly in specialist referral centres. Understanding frequency of adverse events, knowledge, adherence and behaviour of VFR travellers and opinion of health professionals could help standardise recommendations across Europe and reduce the incidence of malaria in VFR travellers.

## Competing interests

The authors declare that they have no competing interests.

## Authors' contributions

GC conceived the study, prepared the original manuscript and coordinated the data collection. GC, RHB, MLS, MPG, FC, JG, ZB, TJ designed and discussed the questionnaires, and discussed the interpretation of results. GC and FG analysed the data. All authors contributed to the drafts, read and approved the final manuscript.

## Supplementary Material

Additional file 1 **Questionnaire part A**. Please answer the following questions, about your experience with immigrants to Europe, returning to their Country of origin to visit friends and relatives ("VFR"). Click here for file

Additional file 2 **Questionnaire part B**. Please find 16 practical cases described below. For every case state if you would recommend chemoprophylaxis (yes/no/uncertain), which chemoprophylaxis (if yes), and possibly why Click here for file
