# Supplementary material for: Malaria chemoprophylaxis recommendations for immigrants to Europe, visiting relatives and friends - a Delphi method study
Source: Malar J. 2011 May 20;10:137. doi: 10.1186/1475-2875-10-137 (PMC3126754; doi:10.1186/1475-2875-10-137)
Supplement: Additional file 1 — Questionnaire part A. Please answer the following questions, about your experience with immigrants to Europe, returning to their Country of origin to visit friends and relatives ("VFR"). [file 1475-2875-10-137-S1.DOC]

**Delphi Malaria – Questionnaire n. 5** **SECTION A**

**Please answer the following questions, about your experience with immigrants to Europe, returning to their Country of origin to visit friends and relatives (“VFR”). You are encouraged to fill in also the “comments” sections**.

1. **What is the average risk perception of malaria in travellers attending your travel clinic?**

- residents in non endemic area ***low***1 2 3 4 5 6 7 8 9 10***high***
- immigrants VFR 1 2 3 4 5 6 7 8 9 10

Comments…………………………………………………………………………………………

1. **What is the accessibility of your travel clinic to these 2 different categories of travellers?**

- residents in non endemic area 1 2 3 4 5 6 7 8 9 10
- immigrants VFR 1 2 3 4 5 6 7 8 9 10

Comments…………………………………………………………………………………………

1. **If a difference is there (question 2), this is due to**

- cost 1 2 3 4 5 6 7 8 9 10
- trust 1 2 3 4 5 6 7 8 9 10
- different perception of the problem 1 2 3 4 5 6 7 8 9 10
- awareness of the service 1 2 3 4 5 6 7 8 9 10
- language problems 1 2 3 4 5 6 7 8 9 10
- locality 1 2 3 4 5 6 7 8 9 10
- other (specify)……………………………………. 1 2 3 4 5 6 7 8 9 10

Comments…………………………………………………………………………………………

1. **What is the proportion of visitors of your clinic who are immigrants returning to their Country of origin to VFR (compared to normal travellers)?**

**………………………%**

1. **Which Region/Country do VFRs attending your clinic originate from?**
   1. ……………………………………………………..
   2. ……………………………………………………..
   3. ……………………………………………………..

Comments…………………………………………………………………………………………

1. **VFRs attend your clinic because of:**

- Malaria risk 1 2 3 4 5 6 7 8 9 10
- Other requirements (e.g. vaccinations) 1 2 3 4 5 6 7 8 9 10
- Previous episode of malaria 1 2 3 4 5 6 7 8 9 10
- other (specify)……………………………………. 1 2 3 4 5 6 7 8 9 10

Comments…………………………………………………………………………………………

1. **Do you alter or adjust your prophylaxis advice for VFR compared to other travellers? (please comment)**

**⁪YES ⁪NO**

Comments…………………………………………………………………………………………

1. **What are your aims in changing prescription for a VFR?**

- Reduce cost 1 2 3 4 5 6 7 8 9 10
- Increase adherence 1 2 3 4 5 6 7 8 9 10
- Take into account semi-immunity 1 2 3 4 5 6 7 8 9 10
- Take into account increased exposure to mosquitoes 1 2 3 4 5 6 7 8 9 10
- Other (specify)…………………………. 1 2 3 4 5 6 7 8 9 10

Comments…………………………………………………………………………………………

1. **After how many years in a non-endemic country you would give to VFRs the same advices you give to non endemic area residents? (please comment)**

**⁪1y ⁪3y ⁪5y ⁪10y ⁪never**

Comments…………………………………………………………………………………………

1. **Which factors do you take into account in prescribing malaria chemoprophylaxis to a VFR? Give your evaluation comparing them on a 1-10 scale (1=minimum account, 10=maximum account).**

- Duration of stay 1 2 3 4 5 6 7 8 9 10
- Area 1 2 3 4 5 6 7 8 9 10
- Itinerary 1 2 3 4 5 6 7 8 9 10
- Likely compliance 1 2 3 4 5 6 7 8 9 10
- Cultural level of the patient 1 2 3 4 5 6 7 8 9 10
- Underlying pathologies 1 2 3 4 5 6 7 8 9 10
- Duration of residence in Europe 1 2 3 4 5 6 7 8 9 10
- other (specify)……………………………………. 1 2 3 4 5 6 7 8 9 10

Comments…………………………………………………………………………………………

1. **How do you evaluate the relevance of a drug’s different attributes, thinking to its use in malaria chemoprophylaxis? Give your evaluation comparing them on a 1-10 scale (1=lowest relevance, 10=highest relevance).**

- Efficacy 1 2 3 4 5 6 7 8 9 10
- Tolerability 1 2 3 4 5 6 7 8 9 10
- Convenience 1 2 3 4 5 6 7 8 9 10
- Causal activity 1 2 3 4 5 6 7 8 9 10
- Cost 1 2 3 4 5 6 7 8 9 10

Comments…………………………………………………………………………………………

1. **Would you alter or adjust your advice to a VFR if accompanied by his/her children born in Europe? (please comment)**

**⁪YES ⁪NO**

Comments…………………………………………………………………………………………

1. **What is the proportion of VFRs in your malaria series?**

**…………………………………%**
