# Supplementary material for: Malaria chemoprophylaxis recommendations for immigrants to Europe, visiting relatives and friends - a Delphi method study
Source: Malar J. 2011 May 20;10:137. doi: 10.1186/1475-2875-10-137 (PMC3126754; doi:10.1186/1475-2875-10-137)
Supplement: Additional file 2 — Questionnaire part B. Please find 16 practical cases described below. For every case state if you would recommend chemoprophylaxis (yes/no/uncertain), which chemoprophylaxis (if yes), and possibly why [file 1475-2875-10-137-S2.DOC]

**Delphi Malaria – Questionnaire n. 5** **SECTION B**

**Please find 16 practical cases described below. For every single case state if you would recommend chemoprophylaxis or not (or if you are uncertain), which chemoprophylaxis (if yes), and possibly why.**

| 1. Male 50 y, from Cameroon, in Europe for 2 years, healthy, travelling to Cameroon for 1 week per month (rural and urban) for years. | | |
| --- | --- | --- |
|  | chemoprophylaxis YES/NO/uncertain | type |
|  | why | |

| 1. Female 20 y, from India (Rajastan), in Europe for 2 years, VFR for 4 weeks, in rural area. | | |
| --- | --- | --- |
|  | chemoprophylaxis YES/NO/uncertain | type |
|  | why | |

| 1. Female 45 y, in Europe for 2 years, VFR in Thailand (Bangkok) for 2 weeks. | | |
| --- | --- | --- |
|  | chemoprophylaxis YES/NO/uncertain | type |
|  | why | |

| 1. Male 30, in Europe for 2 years, VFR in Congo for 12 months, previous intolerance to mefloquine (not well defined). | | |
| --- | --- | --- |
|  | chemoprophylaxis YES/NO/uncertain | type |
|  | why | |

| 1. Male 25, from Guatemala, in Europe for 2 years, travelling for business for 2 months in Mexico, Guatemala, Costa Rica, Panama. | | |
| --- | --- | --- |
|  | chemoprophylaxis YES/NO/uncertain | type |
|  | why | |

| 1. Female, 30, from Thailand, in Europe for 2 years, travelling in Thailand, Cambodia and Vietnam for 2 months, including 5 days in multi-resistance areas. | | |
| --- | --- | --- |
|  | chemoprophylaxis YES/NO/uncertain | type |
|  | why | |

| 1. Male 25, in Europe for 2 years, VFR in south Ivory Coast for 1 month (rural area). | | |
| --- | --- | --- |
|  | chemoprophylaxis YES/NO/uncertain | type |
|  | why | |

| 1. Female 25, in Europe for 2 years, VFR in Ghana for 1 month (rural area), with her 4 years old child, born in Africa, and 1 year old child, born in Europe. | | |
| --- | --- | --- |
|  | 8.1 Chemoprophylaxis (adult) YES/NO/uncertain | type |
|  | why | |
|  | 8.2 Chemoprophylaxis (child 4 y) YES/NO/uncertain | type |
|  | Why | |
|  | 8.3 Chemoprophylaxis (child 1 y) YES/NO/uncertain | type |
|  | Why | |

| 1. Male, 50, in Europe for 2 years, HCV cirrhosis (compensated) VFR in Mozambique for 1 month. | | |
| --- | --- | --- |
|  | chemoprophylaxis YES/NO/uncertain | type |
|  | Why | |

| 1. Female, 50, in Europe for 2 years, VFR in Kenya 1 week in coast area, in January. | | |
| --- | --- | --- |
|  | chemoprophylaxis YES/NO/uncertain | type |
|  | Why | |

| 1. Female, 25, in Europe for 2 years, VFR in Kenya, 1 week in coast area in January, with her children 2 (born in Europe) and 5 years old (born in Kenya). | | |
| --- | --- | --- |
|  | 11.1 Chemoprophylaxis (adult) YES/NO/uncertain | type |
|  | Why | |
|  | 11.2 Chemoprophylaxis (child 2 y) YES/NO/uncertain | type |
|  | Why | |
|  | 11.3 Chemoprophylaxis (child 5 y) YES/NO/uncertain | type |
|  | Why | |

| 1. Female, in Europe for 2 years, 20 years old, 10 weeks pregnant, VFR in Nigeria for 2 weeks (unavoidable travel) | | |
| --- | --- | --- |
|  | chemoprophylaxis YES/NO/uncertain | type |
|  | Why | |
